# Supplementary material for: Peripheral line for vasopressor administration: Prospective multicenter observational cohort study for survival and safety
Source: PLoS One. 2025 Oct 13;20(10):e0333275. doi: 10.1371/journal.pone.0333275 (PMC12517475; doi:10.1371/journal.pone.0333275)
Supplement: Attachment — Submitted filename: Response to reviewers PLVA.docx [file pone.0333275.s002.docx]

**Responses for Editor and Reviewer**

Dear Editor and reviewers, we appreciate the feedback provided to us by the reviewers and editors. The comments actually helped us to improve our manuscript for more scientific rigor. Below are our responses:

1. **Formatting Issues**
   a) Ensure the manuscript follows PLOS ONE's style guidelines.
   b) Rename the file according to PLOS ONE's requirements.

**Response:** the manuscript has been edited to adhere to PLOS ONE style guideline and file renamed according to PLOS ONE’s requirements

2. **Funding Disclosure:** Add a statement about the funders’ role in the study.

**Response:** The funder for our research had no role in study design, data collection or analysis. This is now explicitly stated in the manuscript.

3. **Ethics Statement Placement:** The ethics statement should only be in the Methods section. Remove it from any other parts of the manuscript.

**Response:** The Ethics statement is removed from other sections of the manuscript but the methodology section.

4. **Data availablity:** If data cannot be shared publicly, explain the legal or ethical reasons, specify the restrictions, and provide a contact person for data requests. If data can be shared, upload an anonymized dataset to a public repository and provide a DOI or link and Update the Data Availability Statement accordingly.

**Response:** We have made the anonymized data set available and uploaded it as a separate file. The data availability statement is updated in the manuscript accordingly.

5. **Methodological Concerns**
   a) **Acknowledge that the study lacks a control group and explain this as a limitation.**
   b) **Provide a justification for the sample size, including a power analysis.**
   c) **Address potential confounding factors like ICU care quality and time to vasopressor initiation.**

**Response:**  Our study is one of the first few prospective cohort studies to assess the safety of administering vasoactive medications through peripheral line. Based on case reports and case series of extravasation injuries during administration of the vasoactive medications through peripheral lines in the 1960’s and 1970’s the guidelines recommended central venous lines for administration of vasoactive medications. Even though the evidence on extravasation injuries are case reports and case series, Due to the guideline’s preferential recommendation of central lines no well-designed studies were conducted so far because of ethical concern. Yet, peripheral lines are used for vasopressor administration in low- and middle-income countries due to cost and availability of the central venous catheter. From these settings retrospective studies and small sample prospective studies have shown that extravasation injuries are rare and the complications rate are lower compared to the complications rates reported with central venous line utilization including infection, pneumothorax and bleeding. Considering the guidelines position and the current evidence we designed our study to have the maximum methodologically rigor without raising ethical concern. So, we designed prospective cohort study design with continuous follow up of the patients till they develop the outcome with statistical analysis using survival analysis to make this study the first well designed study to assess the safety of peripheral line for vasopressor administration. We didn’t intervene on the decision of administration via peripheral line but we followed patients for whom vasoactive medications administered via peripheral lines. It would still be ethically challenging to do randomized controlled trial on this title unless this study and other well designed prospective studies are published and the guidelines give similar recommendation for both central and peripheral line use.

Clearly emphasized the lack of control arm limits causal interpretations and put it as a limitation of the study and why randomized controlled trial couldn’t be conducted at this stage. We have also recommended to do randomized controlled studies based on the result of this study.

We have made significant edit to explicitly state confounding factors such as ICU care quality and time to vasopressor initiation might contribute for high septic shock related mortality

6. **Statistical Issues:** Ensure confidence intervals (CIs) are consistently reported for hazard ratios. Include effect size measures to assess the clinical relevance of findings.

**Response:** We have included confidence intervals with the hazard ratio on a variable where it was missing. We have included both crude and adjusted hazard ratios as measures of effect size to quantify the magnitude of differences between groups. Additionally, we have used the Kaplan-Meier survival plot to visually illustrate time-to-event outcomes across groups.

7. **Data Interpretation & Language:** Moderate conclusions regarding the safety of peripheral venous catheter use. Mention that extravasation occurred after 5 days. Improve the clarity of the manuscript, especially in the Methods section

**Response:** We have revised the conclusions to present a more measured interpretation regarding the safety of peripheral venous catheter use. Specifically, we now note that extravasation occurred on the fifth day of catheter placement. Additionally, we have thoroughly revised the Methods section to enhance clarity and ensure that all procedures are described in a clear and reproducible manner.

8. **General Writing & Clarity:** Fix grammatical errors and improve language clarity. Ensure the discussion clearly states what was tested and the study’s limitations.

**Response:** We have carefully reviewed and corrected grammatical errors throughout the manuscript and revised several sections to improve overall clarity. The conclusion part has been edited to make it moderated and the discussion part is edited to better clarify the safe time limit for PVC use.

**Response for Editors On Revision Number Two**

1. Please state what role the funders took in the study. If the funders had no role, please state: "The funders had no role in study design, data collection and analysis, decision to publish, or preparation of the manuscript." If this statement is not correct you must amend it as needed. Please include this amended Role of Funder statement in your cover letter.

**Response:** The role of the funder in the research is clearly stated in the edited manuscript as “The funder had no role in study design, data collection and analysis, decision to publish, or preparation of the manuscript” declaration statement section of the manuscript. The same statement is used in the updated cover letter.

2. Please upload a Response to Reviewers letter which should include a point by point response to each of the points made by the Editor and / or Reviewers. (This should be uploaded as a 'Response to Reviewers' file type.)

**Response:** Response to reviewers and editors is uploaded in a separate .docx file format as requested

Fitsum A. Gemechu, MD
Corresponding Author
