# Supplementary material for: Peripheral line for vasopressor administration: Prospective multicenter observational cohort study for survival and safety
Source: PLoS One. 2025 Oct 13;20(10):e0333275. doi: 10.1371/journal.pone.0333275 (PMC12517475; doi:10.1371/journal.pone.0333275)
Supplement: Attachment — Submitted filename: Response to Reviewers.docx [file pone.0333275.s003.docx]

Dear Reviewers,

We would like to thank you for your thorough review and constructive feedback on our manuscript. Below, we address each of your comments point by point, indicating the corresponding changes made in the revised manuscript.

**Reviewer #1**

1. **Methodological Concerns:** The absence of a control group (e.g., patients receiving vasopressors via central venous catheters) limits the strength of your conclusions. This limitation should be clearly acknowledged and discussed in greater depth. The sample size calculation requires more detailed justification. Please include a formal power analysis to support the adequacy of your study design. The reported mortality rates (57.6% overall and 67.8% in septic shock) are significantly higher than in comparable studies. Potential confounding variables, such as ICU resource levels, severity scores, or timing of vasopressor initiation, should be addressed or discussed as possible explanations.

**Response:**

- We acknowledge that the lack of a control group (e.g., patients receiving vasopressors via central venous catheters) limits direct comparison and causal interpretation. This limitation is now discussed in greater depth in the revised **Limitations of the Study** section, along with an explanation that due to severe resource constraints, central venous catheter insertion is rarely performed in our setting. We have also emphasized that this study can serve as a springboard for future randomized controlled trials.
- Regarding the sample size, we have now included a detailed justification and a post-hoc power analysis in the **Methods** section, indicating a statistical power of 89%, which we consider adequate.
- The higher mortality rates observed (57.6% overall and 67.8% in septic shock) are now explained in the **Limitations** section, highlighting possible confounding factors such as late presentation, ICU resource limitations, baseline illness severity, and delays in vasopressor initiation.

**2. Statistical Analysis: Confidence intervals are not consistently reported for all hazard ratios, limiting interpretation of statistical precision. No effect size measures (e.g., absolute risk reduction, NNT) are provided, which would help assess the clinical significance of your findings. The proportional hazards assumption is briefly mentioned, but no diagnostic plots or formal outputs (e.g., Schoenfeld residual test results) are included. Consider providing these in supplementary materials.**

**Response:**

- We apologize for the oversight. Confidence intervals are now consistently reported for all hazard ratios throughout the manuscript.
- Relative effect size measures such as absolute risk reduction (ARR) and number needed to treat (NNT) could not be calculated due to the absence of a control group.
- We have also included the results of the Schoenfeld residual tests and a summary of the proportional hazards assumption in **Table 4**, with an expanded explanation in the **Statistical Analysis** section.

**3. Data Availability: PLOS ONE requires that data underlying the findings be fully available without restriction. Your current statement indicates data are only available upon request due to privacy concerns. Please consider depositing anonymized data in a public repository or provide a stronger justification aligned with journal policy.**

**Response:**

- We have updated the **Availability of Data and Materials** section in accordance with PLOS ONE guidelines. All data generated and analyzed in this study are now publicly available. The de-identified datasets used for analysis can be accessed at the following repository: [https://doi.org/10.5281/zenodo.16872086](https://doi.org/10.5281/zenodo.16872086). Additionally, similar datasets in .xlsx and .dta formats are attached to this submission for transparency.

**4. Language and Clarity: The manuscript contains grammatical errors, awkward phrasing, and inconsistent use of tense, especially in the Methods section. A thorough language revision by a native English speaker or professional editor is strongly recommended.**

**Response:**

- We acknowledge these language issues. The manuscript has undergone a thorough language revision, focusing particularly on the **Methods** section, to improve grammar, sentence structure, and clarity.

5. **Conclusions and Interpretation: While the study shows a low rate of extravasation (1.2%), all events occurred after prolonged vasopressor use (>5 days). The current conclusion that PVC use is "safe" should be moderated, and a time limit recommendation (e.g., short-term use) should be explicitly discussed. The potential risks of long-term peripheral administration warrant greater emphasis to guide clinicians effectively.**

**Response:**

- We agree with this comment. The **Conclusion** section has been revised to moderate the safety claim, explicitly recommending PVC use only for short-term vasopressor administration. We have highlighted that while the extravasation rate was low (1.2%), all events occurred after more than five days of use, highlighting the need for caution with prolonged administration.

We believe these revisions address the reviewer’s concerns and strengthen the clarity, rigor, and transparency of our manuscript. We thank you again for your valuable feedback.

**Reviewer #3**

1. With respect to data availability, authors stated some limitation, please provide reason and which data are not available.

**Response:**

- As stated above, we have updated the **Availability of Data and Materials** section in accordance with PLOS ONE guidelines. All data generated and analyzed in this study are now publicly available. The de-identified datasets used for analysis can be accessed at the following repository: [https://doi.org/10.5281/zenodo.16872086](https://doi.org/10.5281/zenodo.16872086). Additionally, similar datasets in .xlsx and .dta formats are attached to this submission for transparency.

Sincerely,
Fitsum A. Gemechu
On behalf of all authors
